# Functional analysis of microorganisms and metabolites in the cecum of different sheep populations and their effects on production traits

**DOI:** 10.3389/fmicb.2024.1437250

**Published:** 2024-09-16

**Authors:** Quanlu Meng, Zhixiong Tang, Feifei Yang, Jinping Shi, Ting Liu, Shuru Cheng

**Affiliations:** ^1^College of Animal Science and Technology, Gansu Agricultural University, Lanzhou, China; ^2^College of Biological and Architectural Engineering, Baoji Vocational and Technical College, Baoji, China

**Keywords:** sheep, performance of production, cecum, microorganisms, metabolites

## Abstract

The purpose of this study was to investigate the effects of intestinal microbiota on the growth and production performance of different groups of sheep, focusing on the role of cecal microbiota in regulating intestinal function, enhancing digestion and absorption, and improving feed utilization. The production performance of MG × STH (Mongolia × Small Tailed Han) F1 hybrids and purebred STH (Small Tailed Han) sheep by measuring various factors, including enzyme activities and VFAs (volatile fatty acids), to analyze changes in cecal fermentation parameters across different sheep groups. Metagenomic and metabolomic sequencing combined with bioinformatics to analyze the cecal contents of the two sheep populations. The study findings indicated that the MG × STH F1 hybrids outperformed the purebred STH in terms of body weight, height, oblique body length, and VFAs (*p* < 0.05). Additionally, the MG × STH F1 higher levels of protease and cellulase in the cecum compared to the purebred sheep (*p* < 0.05). Metagenomic analysis identified 4,034 different microorganisms at the species level. Five differential organisms (*Akkermansiaceae bacterium*, *Escherichia coli*, unclassified p Firmicutes, *Streptococcus equinus*, *Methanobrevibacter millerae*) positively regulated sheep performance. Metabolomics identified 822 differential metabolites indoleacetaldehyde, 2-aminobenzoic acid, phenyl-Alanine, enol-phenylpyruvate and n-acetylserotonin were associated with improved performance of sheep. The combined results from the metagenomic and metabolomic studies suggest a positive correlation between specific microbes and metabolites and the performance of the sheep. In conclusion, the MG × STH F1 hybrids demonstrated superior growth performance compared to the purebred STH sheep. The identified microorganisms and metabolites have promising roles in positively regulating sheep growth and can be considered key targets for enhancing sheep performance.

## 1 Introduction

Mongolian (MG) sheep is a special sheep breed in the plateau region of northwest China. It has strong resistance to stress and is of great significance to the life of herdsmen and economic development in the plateau region. Small-tailed Han (STH) sheep, as one of the outstanding sheep breeds in China, has the characteristics of rapid growth and high fecundity, and is one of the main breeds of sheep in China, but STH has drawbacks in environmental stress resistance and feed utilization (Shi et al., 2021). As the most common breeding method in agricultural production, cross breeding is widely used in planting and breeding (Hou and Yi, 2023; Tian et al., 2023). In production practice, Mongolian sheep and small-tailed Han sheep are usually used for cross breeding to produce MG × STH F1 with better performance than their parents in order to improve economic benefits.

Microbes are a class of tiny organisms, including bacteria, fungi, viruses, protozoa, etc. They are widely found in various environments such as soil, water, air and organisms. Microbial fermentation is a widespread biochemical reaction in the metabolic process of microorganisms, which is of great significance in the production of organic acids and other compounds. During fermentation, microorganisms use substrates such as carbohydrates to convert them into specific products, including but not limited to VFAs, alcohols and gasses, through enzymatic catalysis under hypoxic or oxygen-limited conditions (Varghese et al., 2022; Zhang et al., 2024). These products have many biological applications (Hou and Yi, 2023). In the animal digestive system, herbivores obtain nutrients by digests forage material, which contains large amounts of cellulose and other polysaccharides (Muhammad et al., 2021; Wang et al., 2024). Indigestible substrates are converted into easily absorbable organic acids and gasses by microbial fermentation in the cecum of animals to provide energy and nutrients for animals (Miranda et al., 2021). As one of the main products of microbial fermentation, VFAs have important applications in food additives, animal feed and environmental engineering (Ozturk and Gur, 2021). Digestive enzymes play a crucial role in microbial fermentation. They can accelerate the conversion reaction of substrates and improve the yield and purity of products (Du et al., 2023). The cecum is one of the main sites of microbial fermentation and contains a rich microbial community that plays an important role in the digestion and health of herbivores (Ballout and Diener, 2021). It was found that 12% of VFAs were produced by cecum fermentation, and the cecum accounted for 17% of the total cellulose digestion (Dixon and Nolan, 1982). In contrast to the rumen, the cecum will secrete mucin-like substances, and its digestive substrates are mainly some polymers that cannot be digested or absorbed by the forestomach, such as lignin and starch (Godoy-Vitorino et al., 2012). As a part of the hindgut system, the cecum has a weak buffer against substances with large acid and basic properties and a weak ability to maintain cecal pH, which can easily cause cecal dysfunction (Gressley et al., 2011). As a fermentation organ, the malnutrition caused by microbial disorders in the cecum can bring great effects on individual animals, such as inducing abortion of pregnant female domestic animals and reducing the body’s immune function (Feng et al., 2022; Nagata et al., 2020; Wu et al., 2023). Studies on cattle have found that adding appropriate amount of probiotics to forage can improve the microbial community richness of cattle cecum and have a positive effect on the growth and fattening of cattle. This change in performance may be related to its digestive and metabolic functions (Fuerniss et al., 2022; Jing et al., 2022). So far, most of the reports have focused on cattle, and most of them have carried out research on microorganisms and functions in the rumen, but there are few studies on sheep cecum (Mercurio et al., 2021; Trueba-Santiso et al., 2021; Vadaq et al., 2022). The cecum is an important organ of sheep fermentation, and the differences in microbial flora may have a certain impact on the growth and development of sheep. In this study, MG × STH F1 and STH were selected as experimental subjects to evaluate the production performance and cecal fermentation parameters of MG × STH F1 and STH. Using the metagenome and metabolome, the microorganisms and metabolites in the cecum were compared to screen the differential microorganisms and metabolites affecting the performance of sheep. In order to improve the performance of sheep and provide theoretical basis.

## 2 Materials and methods

### 2.1 Animal feeding and sample collection

The experiment was carried out at the Shanhu Breeding Farm in Ganzhou District, Zhangye City, Gansu Province. MG (paternal) × STH (maternal) F1 was used as experimental group, and pure-bred STH was used as control group. Fifty lambs of 1 month ±3 days of age were selected and weaned at 2 months of age under the same conditions of feeding and nutritional requirements (Supplementary Table S1). At 6 months of age, six sheep were randomly selected from the two groups for performance determination and slaughtered (neck bloodletting method, fasting) in Zhangye City; each sample was measured three times. Cecal contents were collected from both groups of animals after slaughter, and content samples (*n* = 6) were randomly selected from both groups to determine the pHs of the contents and subjected to targeted metabolomic, metagenomic, and untargeted metabolomic sequencing. Targeted metabolome was used to analyze VFAs formation in the contents, and metagenomic and untargeted metabolome were used to detect microbial and metabolite identification in the cecal contents. An additional 15 mL of cecal contents were collected from both groups and stored in sterilized containers (*n* = 6, T = −20°C) for the determination of digestive enzyme activities. All samples were collected in accordance with the ethical code approved by the Animal Welfare Committee of College of Animal Science and Technology, Gansu Agricultural University (GAU-AEW-2017-0308).

### 2.2 Determination of pH and fermentation parameters of cecal contents

pHs measurements were performed on the collected cecal contents using a PHS-10 pH meter (ARK.IO, Chengdu, China), and each set of measurements was repeated three times. A 0.2 g sample of cecal contents was placed into a 2 mL centrifuge tube, 1 mL PBS (phosphate buffer solution) solution was added, and homogenized in a high-throughput grinder. After completion, the samples were centrifuged at 10,000 rpm for 3 min at 4°C, and the supernatant was taken for measurement. The activities of β-glucosidase, protease, lipase, α-amylase, and cellulase in cecal contents were measured by colorimetry according to the test method of Zhang et al. (2021) and Teng and Xu (2007).

### 2.3 Detection of VFAs in cecal contents

VFAs in cecal contents were detected using targeted metabolomics. Samples were thawed on ice and 30 M samples were collected in 2 mL centrifuge tubes. Then, 50 μL of 20% phosphoric acid was added and resuspended, and 4-methylvaleric acid (final concentration of 500 μg/mL) was added as an internal standard, and the mixture was shaken and mixed for 2 min. The samples were centrifuged at 14000 g for 20 min, and the supernatant was added to the injection bottle for GC–MS (gas chromatography–mass spectrometry) detection. The injection volume was 1 μL, and the split ratio was 10:1. Samples were separated using an Agilent DB-FFAP capillary column (30 m × 250 μm × 0.25 μm) gas chromatography system. Temperature programming: initial temperature 90°C; The temperature was raised to 160°C at 10°C /min; The temperature was then raised to 240°C at 40°C /min and maintained for 5 min. Helium was used as the carrier gas at a flow rate of 1.0 mL/min. One QC sample was set up for every certain number of experimental samples in the sample cohort to test and evaluate the stability and repeatability of the system. Mass spectrometry was performed on a 5977B MSD (mass selective detector) mass spectrometer (Agilent, California, United States). 5977B MSD conditions are as follows: injection port temperature 250°C; the ion source temperature was 230°C. The temperature of the transmission line was 250°C and the temperature of the quadrupole was 150°C. Electron impact ionization (EI) source, electron energy 70 eV; the test objects were detected by SCAN/SIM (scans/ selected ion monitoring) model. MSD ChemStation software was used to extract chromatographic peak areas and retention times. A standard curve was drawn to calculate the content of short fatty acids in the samples.

### 2.4 Metagenomic sequencing and bioinformatics analysis were performed

DNA was extracted from cecal contents, and the integrity of extracted DNA was checked by 1% agarose gel electrophoresis after completion. The DNA fragments were segmented into approximately 350 bp fragments using a Covaris M220 (Gene Company Limited, Hongkong, China) instrument for PE library construction. Sequencing was performed using the Illumina NovaSeq6000 (Illumina Inc., San Diego, CA, United States) sequencing platform. Adaptor sequences were excised with the use of FAST software (version 0.20.0), along with low-quality reads that were less than 50 bp in length or had a mass value of less than 20 or contained N bases (Li and Durbin, 2010). BWA (version 0.7.9a) was used to compare the reads after quality control with the sheep reference genome to remove the host genome. Sequencing data were assembled using Megahit (Version 1.1.2), and repeat sequences >300 bp in length were selected for gene annotation (Li et al., 2015). Using the Prodigal v2.6.3^1^ for length > 100 bp open reading frames (ORFs) forecast. All samples predicted gene sequences, with CD-HIT software^2^ clustering, each class take the longest sequence of gene as a representative, build the redundant gene set (Li and Godzik, 2006). SOAPaligner (v 2.21) software was used to align the high-quality reads of each sample with the non-redundant gene set, and the abundance information of genes in the corresponding sample was counted. Using DIAMOND software^3^ will not redundant genes set and compare the NR database (than type: BLASTP), and the species annotation was obtained through the corresponding taxonomic information database of the NR library, and then the abundance of the species was calculated using the sum of the abundances of the genes corresponding to the species (Wang and Mu, 2003). Using the corresponding tools of KEGG (v 94.2) and CAZy database (v 5.0), the expected value e-value of alignment parameters was set to 1e^−5^ for relevant annotation, and the total abundance of genes was used to calculate functional and active enzyme fractions.

### 2.5 Metabolomics sequencing and bioinformatics analysis

Fifty mg of cecal contents were added to a 2 mL centrifuge tube and a ground bead 6 mm in diameter was added. 400 μL of the extract [methanol: water =4:1 (v:v)] containing 0.02 mg/mL of the internal standard (L-2-chlorophenylalanine) was used for metabolite extraction. The sample solution was ground on a frozen tissue grinder for 6 min (−10°C, 50 Hz), and then extracted by low temperature ultrasound for 30 min (5°C, 40 kHz). The samples were placed at −20°C for 30 min, centrifuged for 15 min (13,000 g at 4°C), and the supernatant was removed into the injection vial with an inner cannula for analysis. An equal volume of all sample metabolites was mixed to prepare Quality control samples (QC) to investigate the repeatability of the whole analysis process. The instrument platform for this LC–MS (liquid chromatography-mass spectrometry) analysis was the ultra-high performance liquid chromatography-tandem Fourier transform mass spectrometry UHPLC-Q Exactive system (Thermo Fisher Scientific, MA, United States) from Thermo Fisher for cecal contents (Wang et al., 2019; Xie et al., 2019). The raw data of LC–MS were imported into Progenesis QI (Waters Corporation, Milford, United States) for ion integration. Public databases at the same time, the mass spectrum information and metabolic HMDB^4^ and Metlin,^5^ and the self-built database matching, metabolites information. The selection of differential metabolites was based on the variable weight value (VIP) obtained by OPLS-DA model and student’s t test *p* value. Metabolites with VIP > 1 and *p* < 0.05 were identified as differential metabolites. Differences metabolites by KEGG database^6^ for the metabolic pathways of annotation, get the difference of metabolites involved in pathways, and through the Python package (scipy. Stats) pathway enrichment analysis, the biological pathways most relevant to the experimental treatments were obtained by Fisher’s exact test (Ren, 2022).

### 2.6 Data statistics and analysis

SPSS version 26.0 (IBM Corp., Armonk, New York, United States) was used for independent variance t-test and correlation analysis, and data are presented as mean ± standard deviation. Use Omic-Share Tools (2024) and Chiplot61 online website (Li et al., 2023)^7^ related O2PLS analysis and histogram drawing. Metabolite traceability and pathway analysis (MPEA) was performed with MetOrigin (Yu et al., 2022). Statistical plots were drawn using GraphPad Prism 9.5.1 (GraphPad Software, Boston, United States).

## 3 Results

### 3.1 Comparison of growth performance and pH of cecal contents

**Figure 1:**
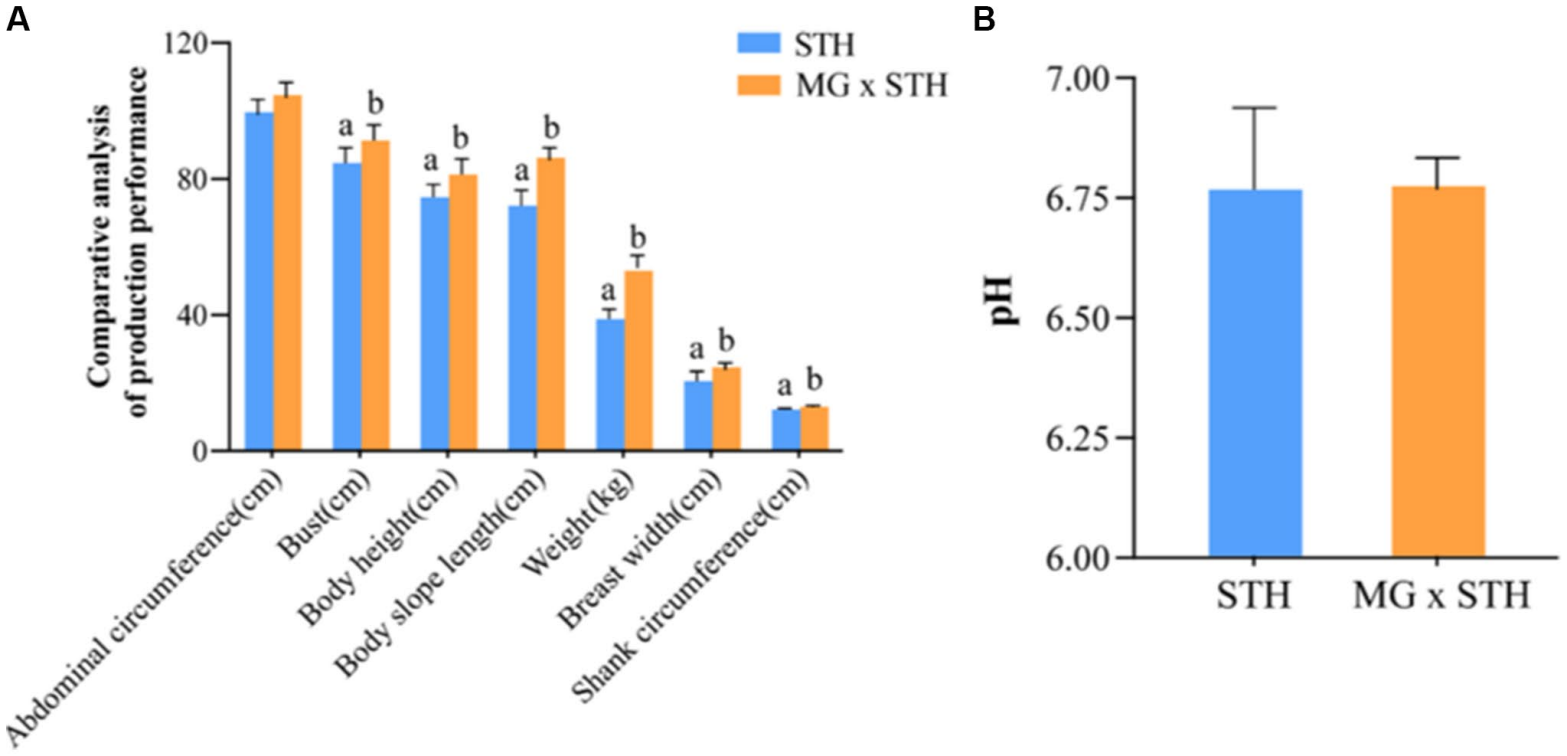
Comparison of production performance and cecal pHs changes between MG × STH F1 and STH. **(A)** The performance of MG × STH F1 and STH was compared using weight, height, body length, chest circumference, tube circumference, chest width and abdominal circumference. **(B)** MG × STH F1 and changes in pHs of STH cecal contents. Different letters indicate significant differences between groups (*p* < 0.05 by independent sample t test).

The production performance of MG × STH F1 and STH was determined (Supplementary Table S2). There were significant differences in weight, height, body length, chest circumference, leg circumference and chest width (*p* < 0.05), but no significant difference in abdominal circumference (*p* > 0.05). The production performance results showed that the production phenotype of MG × STH F1 was superior to STH (Figure 1A).

The results of the pHs assay of the cecal contents indicated that there was no significant difference between the two in MG × STH F1 and STH cecum (Figure 1B; Supplementary Table S3).

### 3.2 Changes of fermentation parameters and digestive enzyme activities of cecal flora

**Figure 2:**
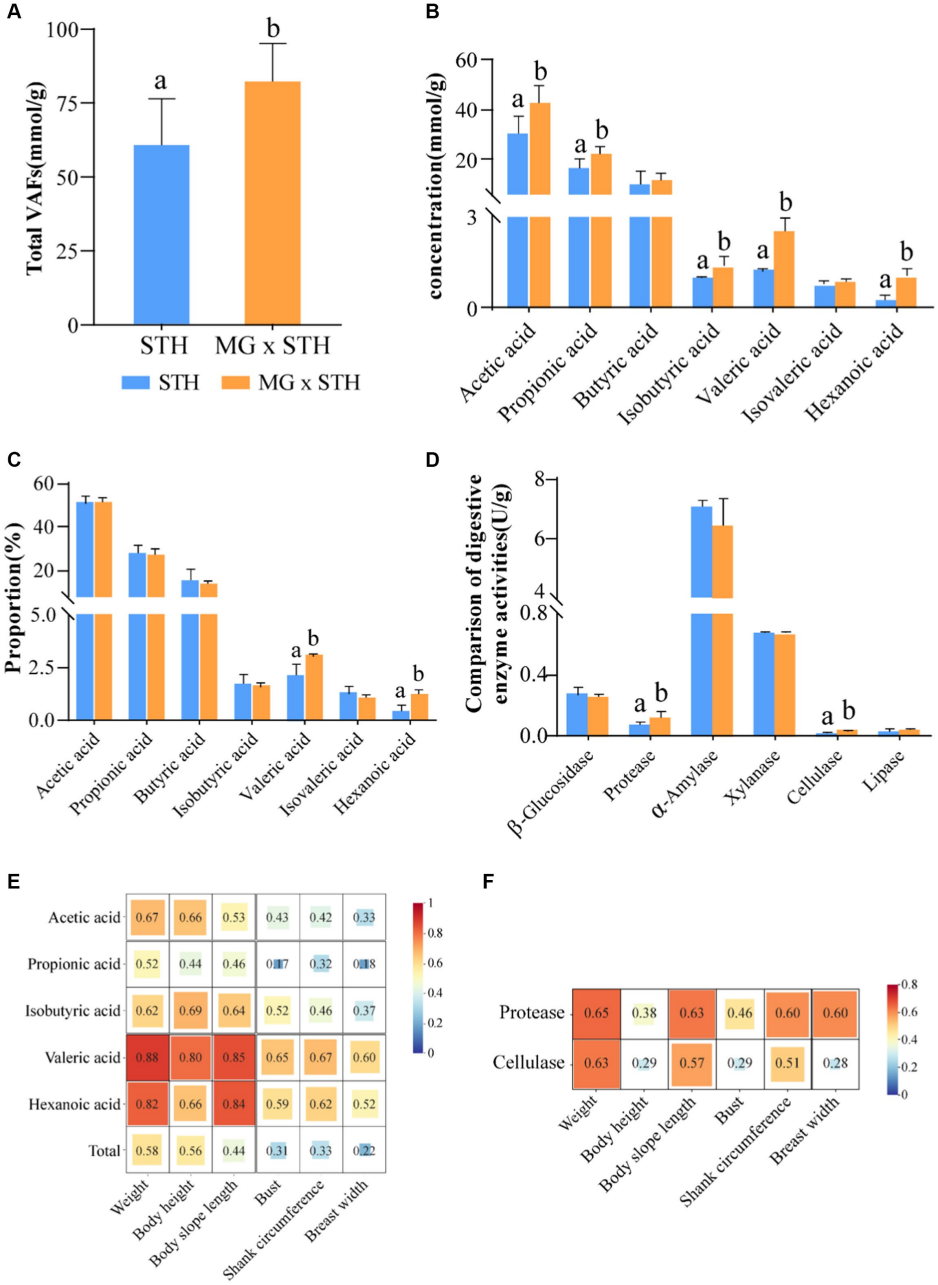
Comparison of fermentation parameters and digestive enzyme activities between MG × STH F1 and STH cecal contents. **(A)** Comparison of total VFAs in MG × STH F1 and STH cecum. **(B,C)** MG× content **(B)** and proportion **(C)** of hexanoic acid, propionic acid, butyric acid, isobutyric acid, valeric acid, isovaleric acid, and hexanoic acid concentrations in STH F1 and STH cecum. **(D)** Comparison of digestive enzymes in MG × STH F1 and STH cecum. **(E,F)** significant differences in the correlation between VFAs and digestive enzyme activities and sheep growth traits. Different letters indicate significant differences between groups (*p* < 0.05 by independent sample t test).

We evaluated seven major VFAs in the cecum as well as changes in fermentation parameters in the intestine. The results showed that the total cecal VFAs were significantly higher in the MG × STH F1 group than in the STH group (*p* < 0.05, Figure 2A; Supplementary Table S4). Furthermore, the concentrations of acetic acid, propionic acid, isobutyric acid, valeric acid, and hexanoic acid in the MG × STH F1 group were higher than in the STH group (*p* < 0.05; Figure 2B). Moreover, the proportion of valeric acid and hexanoic acid increased by 0.91 and 0.98%, respectively, in the MG × STH F1 group compared to the STH group (*p* < 0.05; Figure 2C).

By comparing the activities of five digestive enzymes in MG × STH F1 and STH cecum, it was found that protease and cellulase activities were significantly increased in MG × STH F1 cecum (*p* < 0.05; Figure 2D; Supplementary Table S5). By analyzing the correlation between the cecal VFAs and digestive enzyme activities and the growth performance of sheep, the results showed that significant differences in VFAs and digestive enzyme activities showed a positive correlation with the growth performance of sheep (*r* > 0; Figures 2E,F), among which valerate and hexanoic acid had the strongest correlation with body weight and body oblique length (*r* > 0.8; Figure 2E). There was also a moderate correlation between total VFA and sheep weight and body height (*r* > 0.5). Proteases and cellulases also had moderate correlations with body weight and body slope length (*r* > 0.5; Figure 2F). The above results suggest that the MG × STH F1 cecal fermentation pattern has changed, which may be related to their microbiota perturbation.

### 3.3 Identification of cecal microbial metagenomic profiles

**Figure 3:**
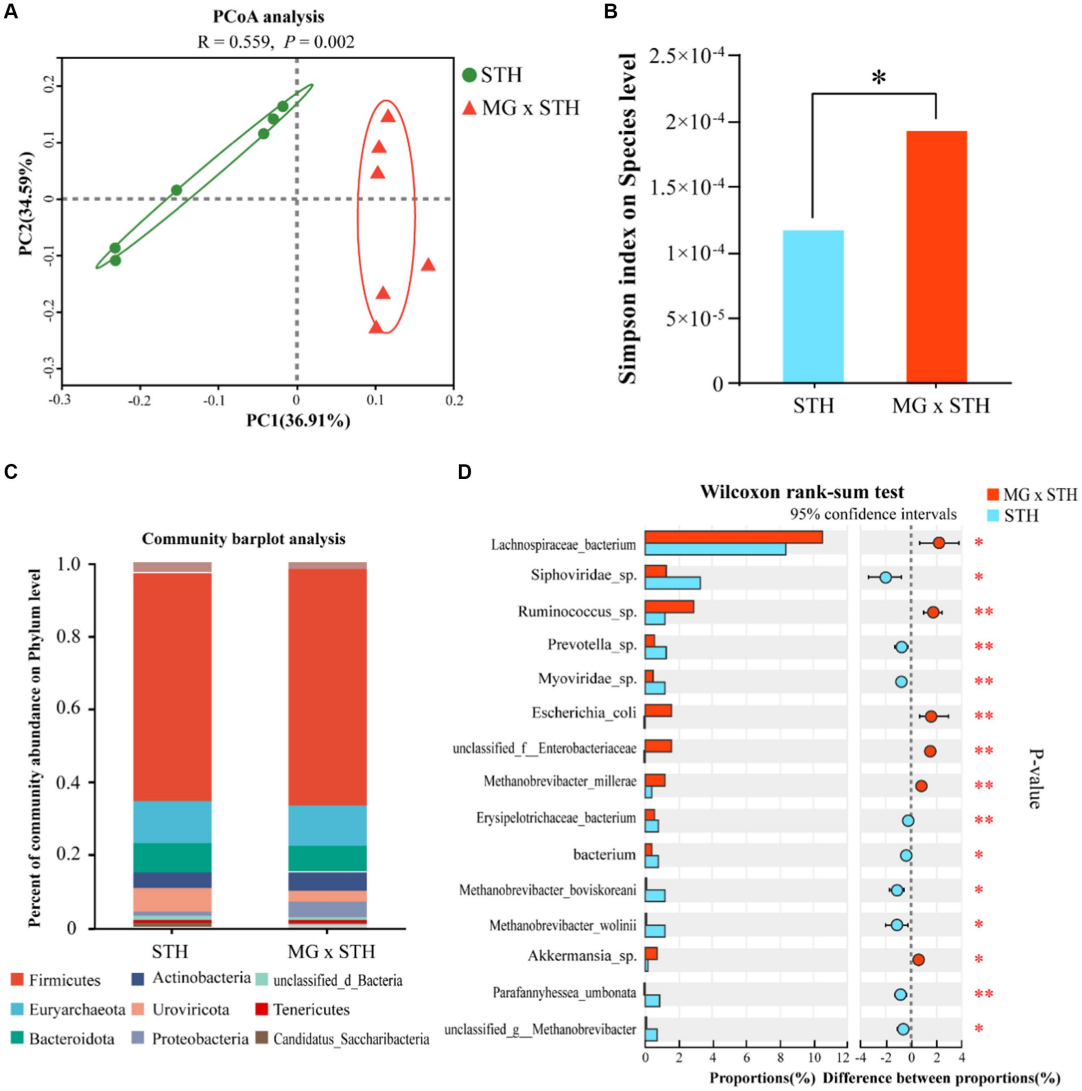
Genus-level PCoA, phyla level microbial composition, and species-level α-diversity. **(A)** Genus-level PCoA analysis. **(B)** Microbial α-diversity test at the species level. **(C)** Dominant flora at phylum level. **(D)** Comparison of STH and MG × STH F1 flora at the species level.

To illustrate that MG × STH F1 cecal microbial diversity had an impact on performance, we performed an analysis of microbial community composition between the two groups based on metagenomic sequencing. The results showed that STH and MG × STH F1 detected an average of 46,934,559 and 45,459,722 raw reads. After the low-quality reads and N-containing reads were cut out, 45,889,530 and 44,434,343 clean reads were obtained from STH and MG × STH F1, respectively. The qualified rates of the two sets of reads were 97.77 and 97.74%, respectively, indicating that the sequencing data were reliable and could be used for subsequent bioinformatics analysis. The structural differences of microbial communities between the two groups were clearly demonstrated based on principal coordinate analysis (PCoA) (Figure 3A). Analysis of the α-diversity of STH and MG × STH F1 cecal flora using the Wilcoxon ranksum test revealed a difference in simpson index between the two groups (*p* < 0.05), but no significant difference in chao and ACE index (*p* > 0.05; Figure 3B; Supplementary Table S6). The difference results between STH and MG × STH F1 showed that the microbial composition in both groups included archaea, viruses, eukaryotes, bacteria and some unclassified microorganisms (Supplementary Figure S1). At the phylum level, the dominant cecum flora in both groups were Firmicutes (STH: 62.55%, MG × STH F1:64.48%), Euryarchaeota (STH: 11.29%, MG × STH F1:11.10%), and Bacteroidetes (STH: 8.09%, MG × STH F1: 7.07%; Figure 3C). Analysis of the differential abundance of STH and MG × STH F1 at the genus level showed that among the top 15 genera with significant changes in abundance, 10 genera were significantly more abundant in the STH group than MG × STH F1, and 5 genera were lower than MG × STH F1 (*p* < 0.05; Supplementary Table S7; Supplementary Figure S2).

### 3.4 Identification and difference comparison of cecal microbiome functional profiles

**Figure 4:**
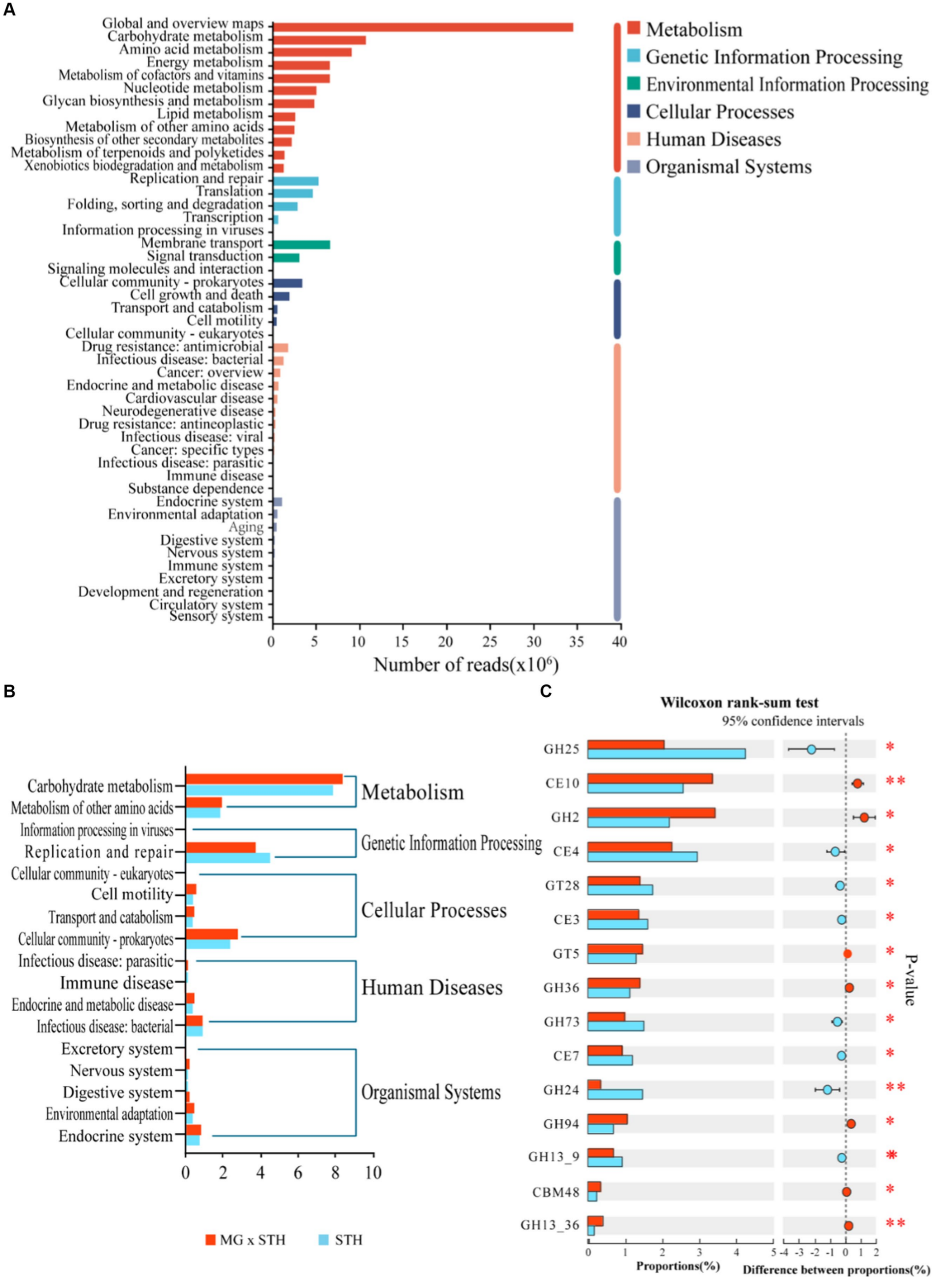
KEGG enrichment analysis results of STH group and MG × STH F1 group. **(A)** First-and second-level enrichment results. **(B)** Significantly enriched pathways at the second level. **(C)** CAZyme expressing top 15 relative abundance.

The functions of cecal microorganisms were identified by KEGG database and CAZyme database. In the KEGG analysis, a total of 6 pathways were annotated at the first level, namely “metabolism,” “genetic information process,” “environmental information process,” “cellular process,” “human disease” and “tissue system” (Figure 4A; Supplementary Table S8). At the second level, KEGG enriched 47 pathways, among which carbohydrate and water metabolism, amino acid metabolism, energy metabolism, cofactor and vitamin metabolism, membrane transport and replication and repair were the most abundant (Figure 4A; Supplementary Table S8), and 17 of them were significantly different (*p* < 0.05; Figure 4B). In particular, MG × STH F1 had significantly higher carbohydrate and water metabolism pathways, cell community-prokaryotes, and environmental adaptability than STH. These results indicate that MG × STH F1 has a higher capacity of carbon and water metabolism than STH, and the retained Mongolian sheep have a strong ability to adapt to the environment. Four hundred and nineteen pathways were enriched in KEGG level 3, of which 148 were statistically significant (*p* < 0.05), including 60 “metabolism,” 11 “genetic information process,” 7 “environmental information process,” 8 “cellular process,” 48 “human disease” and 14 “tissue system” (Supplementary Table S8). The CAZyme map identified a total of 573 genes encoding CAZyme, of which 143 were statistically significant (*p* < 0.05; Supplementary Table S9). It includes 5 accessory activities (AA), 15 carbie-binding modules (CBMs), 8 car-bohydrate esterase (CE), and 5 accessory activities (AA). Sixty-four glycoside hydrolases (GH), 30 glycosyltransferases (GT) and 21 polysaccharide lyases (PL), but no PL was found in the top 15 of relative abundance of expression (Figure 4C). Of these CAZymes involved in carbohydrate metabolism, 19 are specifically expressed in MG × STH F1 (1 AA, 3 CBM, 7 GH, 5 GT, and 3 PL) and 8 are specifically expressed in STH (3 GH, 1 GT, and 4 PL). The remaining CAZyme was expressed in both groups, and the expression levels were significantly different.

### 3.5 Cecal metabolomic analysis

**Figure 5:**
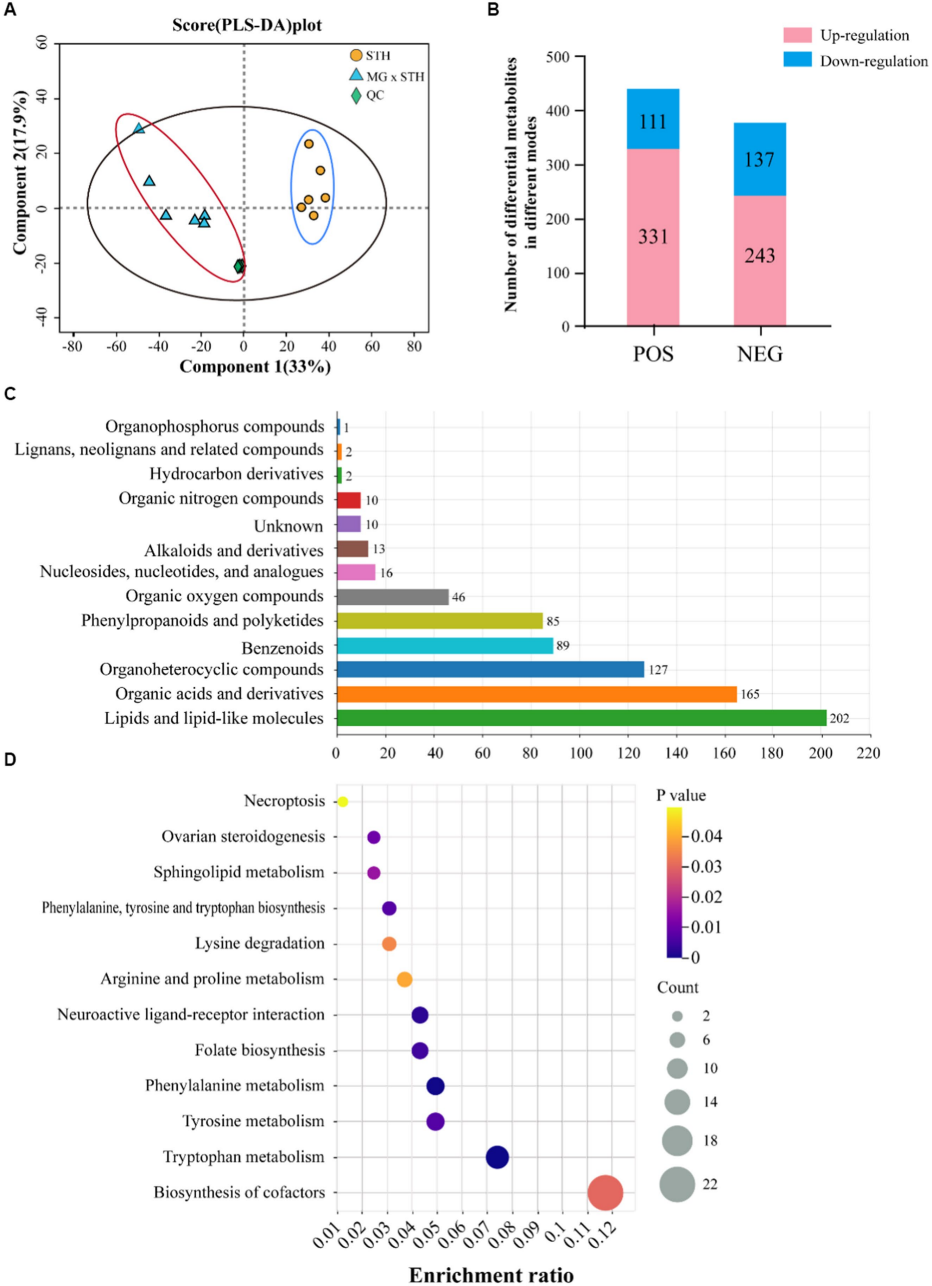
Results of STH group and MG × STH F1 untargeted metabolome assays. **(A)** Results of STH group and MG × STH F1 PLS-DA analysis. **(B)** The number of differential metabolites detected in different ion modes and their up-or down-regulation. **(C)** Differential metabolite classification. **(D)** KEGG signaling pathways enriched for differential metabolites.

Metabolites in MG × STH F1 and STH cecum were identified by LC–MS, and PLS-DA results showed large differences in metabolite species and content between the two groups (Figure 5A; Supplementary Figure S3). A total of 822 differential metabolites were identified in the two modes (*p* < 0.05, VIP > 1), of which 442 were identified in the positive mode (Up-regulated 331, Down-regulated 111) and 380 were identified in the negative mode (Up-regulated 243, Down-regulated 137, Figure 5B; Supplementary Table S10). These metabolites were annotated by HMDB, and 768 metabolites were able to be annotated to the information. Classification of the annotated metabolites showed that, Lipids and lipid-like molecules (202 species) contained the most metabolites, followed by Organic acids and derivatives (165 species) and Organoheterocyclic compounds (127, Figure 5C; Supplementary Table S11). KEGG functional annotation results showed that 149 differential metabolites were annotated by 30 KEGG pathways at level 2, including metabolic processes such as base acid metabolism, lipid metabolism and carbohydrate metabolism (Supplementary Figure S4). KEGG pathway enrichment analysis of differential metabolism annotated to the information showed that a total of 93 pathways were enriched, of which 12 were statistically significant (*p* < 0.05), including cofactor synthesis and multiple amino acid metabolism pathways (Figure 5D).

### 3.6 Combined metagenomic and metabolomic analyses

**Figure 6:**
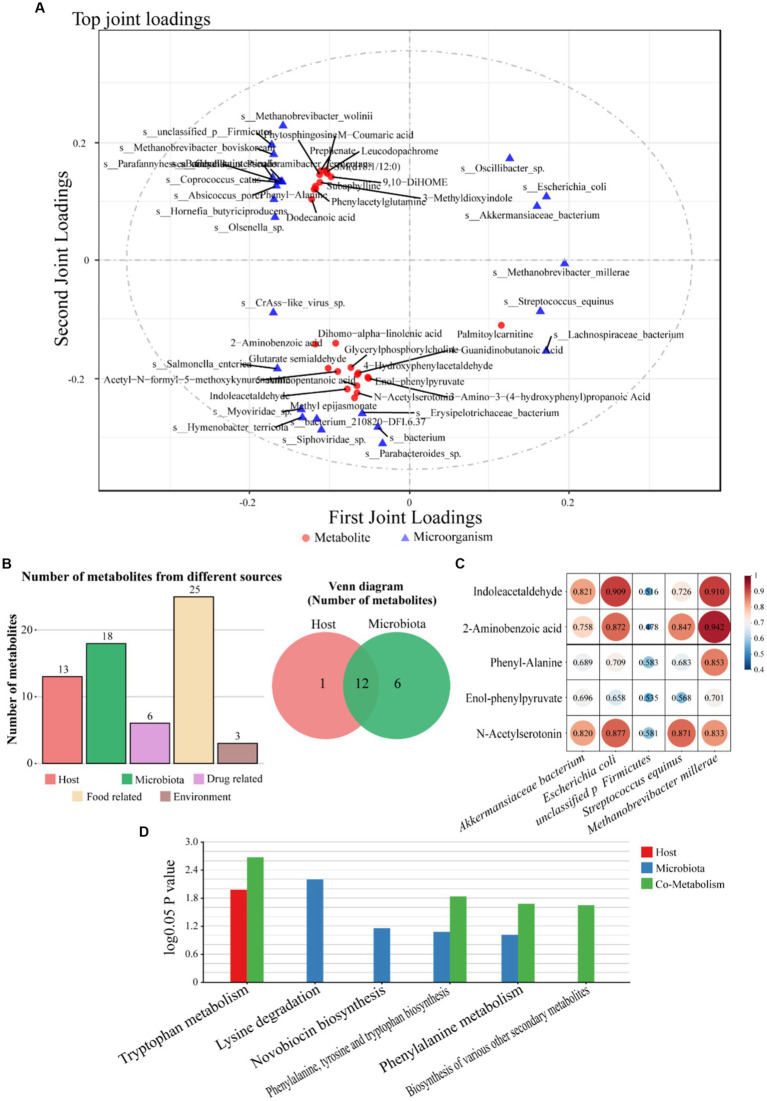
Combined metagenomic and metabolome analysis. **(A)** Metagenomic and metabolomic O2PLS analysis. **(B)** Top 25 metabolites MPEA analysis, left: metabolite origin analysis, right: host and microbial origin Venn analysis. **(C)** Correlation analysis between target metabolites and microorganisms. **(D)** Microbial and metabolite enrichment results are displayed.

To determine the influence of cecal microbes and metabolites on sheep growth performance, the presence of linkage effects between microbes produced by the metagenome and metabolites produced by the metabolome was tested. The microorganisms and metabolites involved in carbohydrate metabolism, energy metabolism, lipid metabolism, amino acid metabolism and other amino acid metabolism in the KEGG pathway enrichment results of the two omics were jointly analyzed. O2PLS test revealed the top 25 microorganisms and metabolites with the largest linkage effects, including 19 bacteria, 3 viruses and 3 archaea. Of these, 10 belonged to the phylum Firmicutes, 4 to the phylum Verrucomicrobacter, 3 to the phylum Euryarchaeota, and the remaining 8 to the phylum Actinobacteria, Proteobacteria, Bacteroidetes, and unknown bacteria (each containing two microorganisms; Figure 6A). The 25 microorganisms and metabolites with the largest linkage effects obtained for O2PLS were further screened for microorganisms associated with metabolites by MPEA analysis with MetOrigin. This analysis was able to classify metabolites from host, food, microbes, and other sources, and MPEA results showed that 18 of the 25 metabolites were microbial-derived, 13 were host-derived, and 12 were shared by both microbes and hosts (Figure 6B). The results of functional enrichment of these metabolites showed that there were 4 enrichment pathways in which microorganisms and hosts participated together (Figure 6D). There were 5 differential metabolites (indoleacetaldehyde, 2-aminobenzoic acid, phenyl-alanine, enol-phenylpyruvate and n-acetylserotonin), and these metabolites were all up-regulated. Eight microorganisms (*Akkermansiaceae bacterium, Escherichia coli*, unclassified p Firmicutes, *Streptococcus equinus*, *Oscillibacter* sp., *Methanobrevibacter millerae*, *Parabacteroides* sp., *Erysipelotrichaceae bacterium*) with FC > 2 were selected from the top 25 differential microorganisms, among which the abundance of *Oscillibacter* sp., *Parabacteroides* sp. and *Erysipelotrichaceae bacterium* were all down-regulated in MG × STH F1.

It is noteworthy that the expression abundance of *Akkermansiaceae bacterium*, *Escherichia coli* and unclassified p Firmicutes in MG × STH F1 was up-regulated 1,086-fold, 396-fold and 390-fold, respectively. To further understand the relationship between the five metabolites and the five up-regulated microorganisms, Pearson correlation analysis was performed, and the results showed that there were positive correlations between the five metabolites and the five microorganisms (Figure 6C).

### 3.7 Correlations among microbes, metabolites, and phenotypes

**Figure 7:**
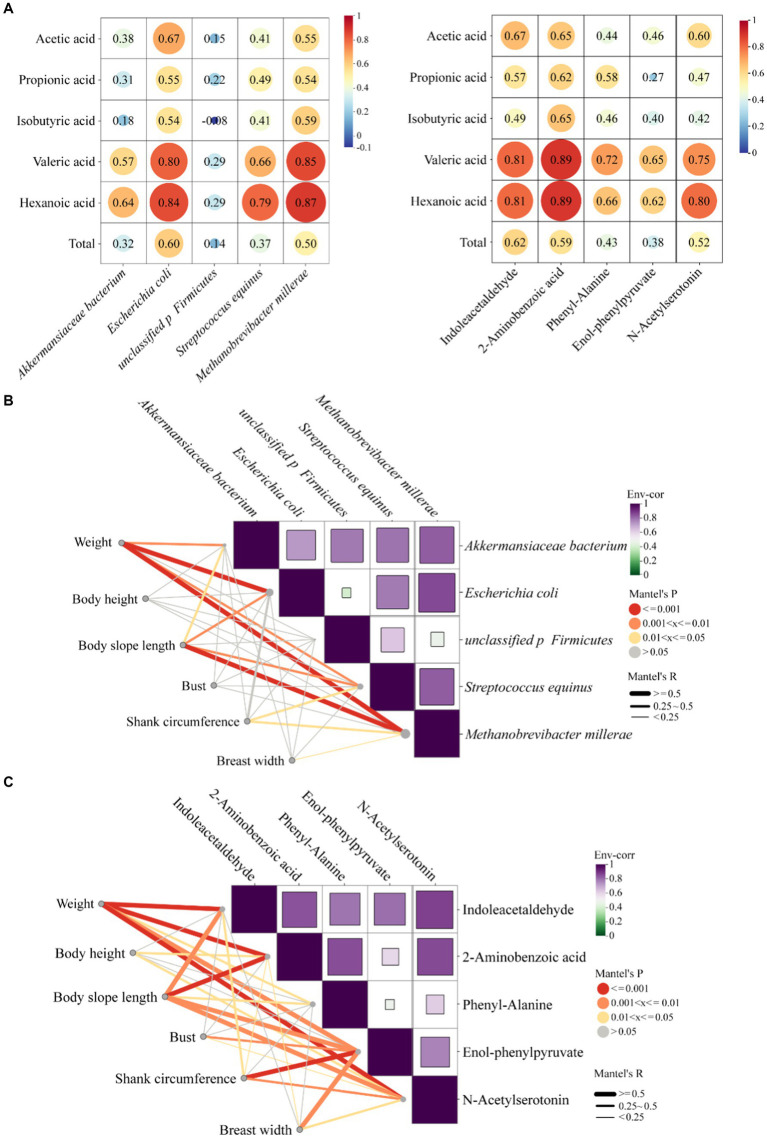
Correlation analysis between microorganisms, metabolites and VFAs and production phenotypes. **(A)** Left: correlation analysis between microorganisms and VFAs, right: correlation analysis between metabolites and VFAs. **(B)** Mantel analysis between microbes and phenotypes. **(C)** Mantel analysis between metabolites and phenotypes.

In order to understand the effects of five microorganisms and five metabolites screened on the growth performance of MG × STH F1 sheep, the correlation between them was analyzed. The heat map of correlation between microorganisms and VFAs showed that five up-regulated microorganisms were positively correlated with VFAs except isobutyric acid and unclassified p Firmicutes. Valeric and hexanoic acid showed the strongest correlations with *Escherichia coli* and *Methanobrevibacter millerae* (*r* > 0.8), and acetic acid, the most abundant VFA in the cecum, also showed moderate correlations with both species (*r* ≥ 0.55, Figure 7A left). The results of correlation between metabolites and VFAs showed that five metabolites were positively correlated with VFAs, indoleacetaldehyde, 2-aminobenzoic acid and n-acetylserotonin were most strongly correlated with valeric acid and hexanoic acid (*r* ≥ 0.75, Figure 7A right). The correlation between these three metabolites and acetic acid was consistent with that of microorganisms (*r* ≥ 0.6).

Mantel analysis was used to test the variation trend and production phenotype differences of five microorganisms and five metabolites. The results showed that *Akkermansiaceae bacterium*, *Escherichia coli*, *Streptococcus equinus* and *Methanobrevibacter millerae* were positively correlated with body weight and body slope length (*r* > 0.25, *p* < 0.05; Figure 7B). *Escherichia coli* and *Methanobrevibacter millerae* were moderately positively correlated with body weight (*r* > 0.5), and *Methanobrevibacter millerae* was moderately correlated with body slope length (*r* > 0.5). There was no significant correlation between body height and. bust and the five microorganisms (*p* > 0.05). The correlation between metabolites and production phenotype showed that there was a positive correlation between five metabolites and production phenotype (*r* > 0.25; Figure 7C), and there was a moderate positive correlation between body weight and the change trend of five metabolites (*r* > 0.5). Body slope length was moderately positively correlated with four metabolites except phenyl-alanine (*r* > 0.5). Body height and bust were also correlated with metabolites, but the overall correlation was weak.

## 4 Discussion

By combining the cecum metagenome and metabolome, the effects of microorganisms and metabolites on growth performance of MG × STH F1 and STH cecum ecological environment changes were analyzed. The effects of related microorganisms and metabolites on VFAs in the cecum were also evaluated. As crucial substances in the growth and development of ruminants, VFAs are produced by microbial fermentation of cellulose and other non-starch polysaccharides in the digestive tract, and play a role in energy supply, nutrient absorption, pH regulation, and growth promotion (Bhagat et al., 2023; Malmuthuge et al., 2019). Through the detection of VFAs in the cecal contents of different sheep groups in this study, it was found that the total VFAs content in MG × STH F1 cecal was significantly higher than STH, and the content of acetic acid, propionic acid and isobutyric acid was significantly different. The analysis of the proportion of these VFAs found that only valeric acid and caproic acid had a difference in the percentage content, indicating that the growth trait of MG × STH F1 was superior to STH due to the VFAs content in the digestive tract. VFAs as organic acids can also regulate the pH change in the gut (Kim and Sung, 2022), but the pH change in the cecum of the two groups of sheep in this study was not significant, which may be related to their microbial adaptability or microbial adaptation. In addition to producing VFAs by fermentation, gastrointestinal microorganisms can also affect the host’s absorption and utilization of nutrients by secreting or regulating the activities of digestive enzymes in the gastrointestinal tract (Krol et al., 2023). In addition, protease and cellulase contents were also significantly higher in MG × STH F1 cecum than in STH. Cellulose content in typical forages (ryegrass, alfalfa and straw of grasses, etc.) is more than 1/5 of the total dry matter, but cellulose has a negative effect on apparent digestibility, mainly because of the lack of enzymes in the digestive system to catabolite cellulose (Akdeniz et al., 2019; Ronn et al., 2022). Cellulases catabolize cellulose and hemicellulose to produce glucose, oligosaccharides and oligosaccharides. Due to the lack of glucose transporter (GLUT) in the cecum, these small molecular sugars are used as the main carbon source by microorganisms in the cecum (Ilic et al., 2023). Although proteases in the cecum play a relatively limited role, they may still play some role in the degradation of some proteins and in the balance of the microbial community. Some studies have shown that the addition of proteases to the diet increases the abundance of microorganisms in the phylum Firmicutes, Bacteroidetes, and Porphyromonas (Huyan et al., 2022).

The metagenomic analysis of MG × STH F1 and STH cecum showed that bacteria were the most abundant microorganism in sheep cecum, accounting for about 85.28% of the total microorganisms, followed by archaea (12.65%) and viruses (5.77%), which were similar to the results of previous studies on other ruminant cecum microorganisms (Morgavi et al., 2013; Shi et al., 2023). At the same time, the microbial differences between MG × STH F1 and STH were mainly reflected in the bacterial level, and it was speculated that the sheep production traits were related to these different bacteria. For ruminants, after the feed enters the rumen, microorganisms complete the initial fermentation and produce VFAs (Ozturk and Gur, 2021), and the undigested food residues enter the digestive tract in the middle and back of the cecum. CAZymes produced by these microorganisms are specific enzymes involved in carbohydrate metabolism, including the degradation, synthesis and modification of cellulose, galactose, xylan and other polysaccharides, which help microorganisms to utilize carbon and energy sources in the environment (Liang et al., 2023; Thapa et al., 2023). CAZyme enrichment results showed that there were significant differences in GH, CE and GT enzymes in the cecum of the two groups of sheep. The overall abundance level of GH in MG × STH F1 was higher than that in STH F1, indicating that MG × STH F1 had better catabolic ability to digester in the cecum, but the abundance trend of GH25 was opposite. This may be related to the fact that α-keto-uronic acid is the main catalytic substrate of GH25, while galactose and cellulose are the main catalytic substrates of other GHS (GH2, GH5, GH36, etc.)(Schwab et al., 2010; Zhou et al., 2014). These CAZyme can result in higher feed conversion and more production of VFAs, which in turn provide more energy supply for host growth and development.

Although the dominant microbiota in the cecal of sheep did not change between the two groups, the results of microbial function enrichment showed that the microorganisms related to environmental adaptability in MG × STH F1 were significantly higher than those in STH, indicating that MG × STH F1 inherited the characteristics of strong stress resistance of Mongolian sheep. *Akkermansiaceae bacterium*, belonging to Verrucomicrobiota, can “feed” on mucin secreted by the host and use mucin as the sole carbon and nitrogen source to produce VFAs such as acetic acid and propionic acid during metabolism (Derrien et al., 2004; Van Herreweghen et al., 2017). *Akkermansiaceae bacterium* can also activate the secretion of gastrointestinal hormone glucagon-like peptide-1 (GLP-1) in the intestine, participate in the regulation of glucose and lipid metabolism, and promote the absorption and utilization of energy substances in the intestine (Yu et al., 2022). Metagenomic results showed that *Akkermansiaceae bacterium* was more than 1,000 times more abundant in MG × STH F1 cecum than in STH, which was hypothesized to be related to the production of VFAs and uptake of energetic substances in MG × STH F1 cecum. Further studies should be done to determine whether the increased abundance of *Akkermansiaceae bacterium* in MG × STH F1 is related to the increased levels of protease. *Escherichia coli* is the most dominant and abundant bacteria in human and animal intestines. It is not directly involved in the fermentation process of ruminants’ digestive tract, but it is related to carbohydrate metabolism, amino acid metabolism and lipid metabolism in organisms (Carreon-Rodriguez et al., 2023; Tsukimi et al., 2024). *Escherichia coli* uses carrier proteins such as polysaccharide channel proteins such as LamB and glycokinase on the outer membrane and inner membrane of the cell to take up foreign sugar molecules (Kim et al., 2022). Sugar molecules that enter *Escherichia coli* are first phosphorylated to form phosphorylated sugar molecules to increase their intracellular stability. These phosphorylated sugar molecules are then further metabolized and are broken down into simpler metabolites such as pyruvate and acetone via the glycolytic pathway (Carreon-Rodriguez et al., 2023). *Methanobrevibacter millerae* can convert H_2_ and CO_2_ in the cecum to CH_4_ using microbial fermentation. Wei et al., cultured five methanogens in the rumen of Qinghai yak and detected their digestion of neutral detergent fiber in straw forage (Wei et al., 2022). The results showed that these methanogens could decompress cellulose to produce CH_4_, acetate and FA. However, as a strong and effective greenhouse gas, CH_4_ emission has a certain impact on global climate change.

The analysis found that there was a positive regulatory relationship between five metabolites and microorganisms, and these metabolites were positively regulated with sheep production phenotype. These five metabolites are mainly related to phenylalanine and tryptophan metabolism (Bose et al., 2024; Fitzpatrick, 2023). Phenyl-alanine, as one of the essential amino acids in animals, is essential for cell growth and metabolism. Enolpyruvate is an intermediate product in the phenyl-alanine metabolic pathway, which is converted from phenyl-alanine by enzymatic reaction and can be further metabolized to other compounds such as other amino acids or pyruvate (Jiang et al., 2023; Shehata et al., 2024). The increased levels of phenyl-alanine and enol-phenylpyruvate in the cecum of MG × STH F1 may be related to *Escherichia coli* because *Escherichia coli* can convert xylose and glucose to phenyl-alanine, In turn, large amounts of enol-phenylpyruvate are produced (Liu et al., 2021). Indoleacetaldehyde is an important compound in plants, mainly acting as a precursor of the plant growth hormone indoleacetaldehyde (IAA), which is converted to IAA through a series of enzymatic reactions, thereby regulating plant growth and development (Cohen et al., 2022). Some studies have identified the association between biomarkers in cattle fecal and marbling standard longissimus dorsi muscle by using non-targeted LC–MS metabolomics and found that Indoleacetaldehyde can be used as a candidate marker closely related to high-grade beef marbling production, the specific mechanism of action has not been explained (Chen et al., 2022). In this study, Indoleacetaldehyde was significantly correlated with the weight, body slant length and shank circumference of sheep (*r* > 0.25, *p < 0.05*), indicating that indoleacetaldehyde did have an effect on the production phenotype of sheep, but the specific mechanism needs to be further studied. 2-Aminobenzoic acid is an aromatic compound belonging to the derivative of amino acids and benzoic acids, and most microorganisms have the ability to synthesize 2-Aminobenzoic acid through the shikimate and folate pathways (Wegkamp et al., 2007). Shikimate is involved in the metabolism of phenylalanine and tryptophan, which can be converted into biologically active substances (such as neurotransmitters) and intermediates involved in energy metabolism. Meanwhile, folate plays a key role in cell division and tissue growth, ensuring that cells can efficiently undergo energy metabolism (Roux and Walsh, 1992). Among these 2-Aminobenzoic acid-producing microorganisms, *Escherichia coli* is the most commonly used engineering bacteria in synthetic biology and metabolic engineering, and the production of 2-Aminobenzoic acid can be effectively increased by constructing high-yielding strains of *Escherichia coli* (Koma et al., 2014). In this study, the metagenomic and metabolomic results showed that *Escherichia coli* and 2-Aminobenzoic acid in MG × STH F1 cecum were significantly higher than those in STH (*p* < 0.05), which was inferred to be caused by the increase of *Escherichia coli* and the increase of 2-Aminobenzoic acid. The synthesis of N-Acetylserotonin is derived from tryptophan, which is first converted to 5-hydroxytryptophan (5-HTP) and then further converted to serotonin 5-Hydroxytryptamine (5-HT), which is acetylated to form N-Acetylserotonin (Kang et al., 2023). In the brain, it is involved in behavioral, cognitive, and motor activation as a neurotransmitter, and in the gut, it affects perisis, motility, mucus secretion, vasodilatation, and nutrient absorption (Correale et al., 2022; Kang et al., 2023). Meanwhile, animal studies have shown that N-Acetylserotonin can alleviate the inflammation of the hindgut, thereby effectively improving the health of the hindgut (Kang et al., 2023).

In summary, these microorganisms and metabolites may affect the intestinal health status and energy metabolism of sheep through phenylalanine metabolism and tryptophan metabolism, thereby improving the performance of sheep.

## 5 Conclusion

The aim of this study was to elucidate taxonomic features, functions, and metabolites of cecal microbes in MG × STH F1 and STH populations, as well as interactions with host growth metabolites. The results of this study showed that MG × STH F1 growth performance and cecal fermentation parameters were superior to STH. Metagenomics and metabolomics identified five growth-promoting microorganisms (*Akkermansiaceae bacterium*, *Escherichia coli*, unclassified p Firmicutes, *Streptococcus equinus* and *Methanobrevibacter millerae*) and five metabolites (indoleacetaldehyde, 2-aminobenzoic acid, phenyl-Alanine, enol-phenylpyruvate and n-acetylserotonin). Furthermore, we found that *Akkermansiaceae*, as a low-abundance microorganism, may play a role in improving sheep performance by regulating metabolites.

## Data Availability

The datasets presented in this study can be found in online repositories. The names of the repository/repositories and accession number(s) can be found in the article/Supplementary material.

## Footnotes

^1^https://github.com/hyattpd/Prodigal

^2^http://www.bioinformatics.org/cd-hit/

^3^http://ab.inf.uni-tuebingen.de/software/diamond/

^4^http://www.hmdb.ca/

^5^https://metlin.scripps.edu/

^6^https://www.kegg.jp/kegg/pathway.html

^7^https://www.chiplot.online/

## Supplementary material

SUPPLEMENTARY FIGURE S1 Annotation of microbial species at the Domain level.

SUPPLEMENTARY FIGURE S2 Microorganisms that differed between groups at the genus level.

SUPPLEMENTARY FIGURE S3 Results of metabolite replacement test.

SUPPLEMENTARY FIGURE S4 KEGG pathway level 2 signaling pathway enriched by metabolites.

SUPPLEMENTARY TABLE S1 Feed formulation for sheep during aquaculture and fattening.

SUPPLEMENTARY TABLE S2 Phenotypic data on growth and development of two sheep populations.

SUPPLEMENTARY TABLE S3 pH of cecal content.

SUPPLEMENTARY TABLE S4 Cecal contents VFAs targeted metabolome results.

SUPPLEMENTARY TABLE S5 Fermentation parameter detection.

SUPPLEMENTARY TABLE S6 Analysis of metagenomic microbial alpha diversity.

SUPPLEMENTARY TABLE S7 Annotation of microbial NR classification.

SUPPLEMENTARY TABLE S8 Microbial KEGG classification.

SUPPLEMENTARY TABLE S9 CAZyme enrichment statistics.

SUPPLEMENTARY TABLE S10 Details of cecal metabolites differences between the two group.

SUPPLEMENTARY TABLE S11 Annotation and classification of metabolites.

SUPPLEMENTARY TABLE S12 KEGG enrichment results of metabolites.
