# Supplementary figures and images for: Functional analysis of microorganisms and metabolites in the cecum of different sheep populations and their effects on production traits
Source: Front Microbiol. 2024 Sep 16;15:1437250. doi: 10.3389/fmicb.2024.1437250 (PMC11439670; doi:10.3389/fmicb.2024.1437250)

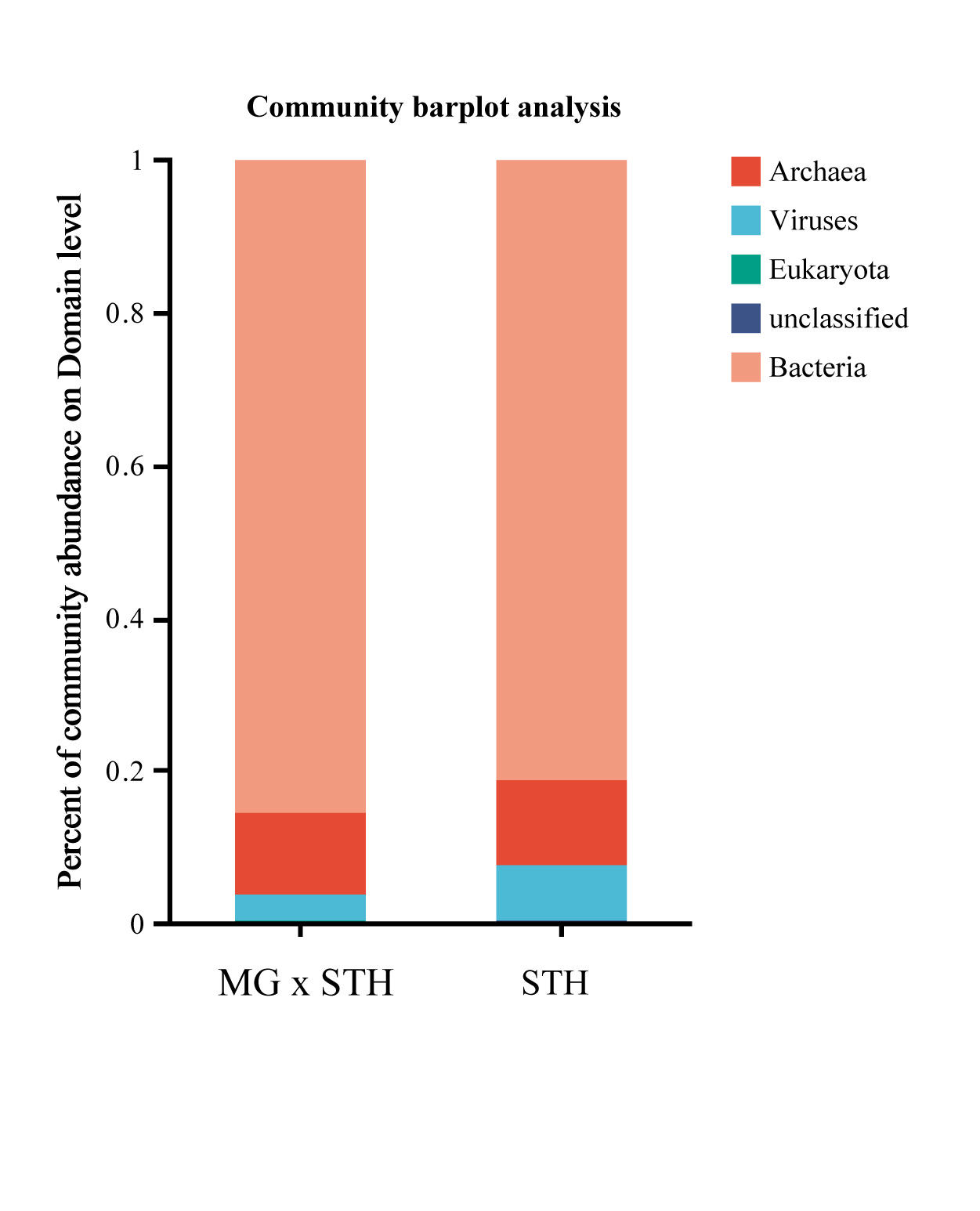

Supplement: SUPPLEMENTARY FIGURE S1 — Annotation of microbial species at the Domain level. [file Image_1.TIF]

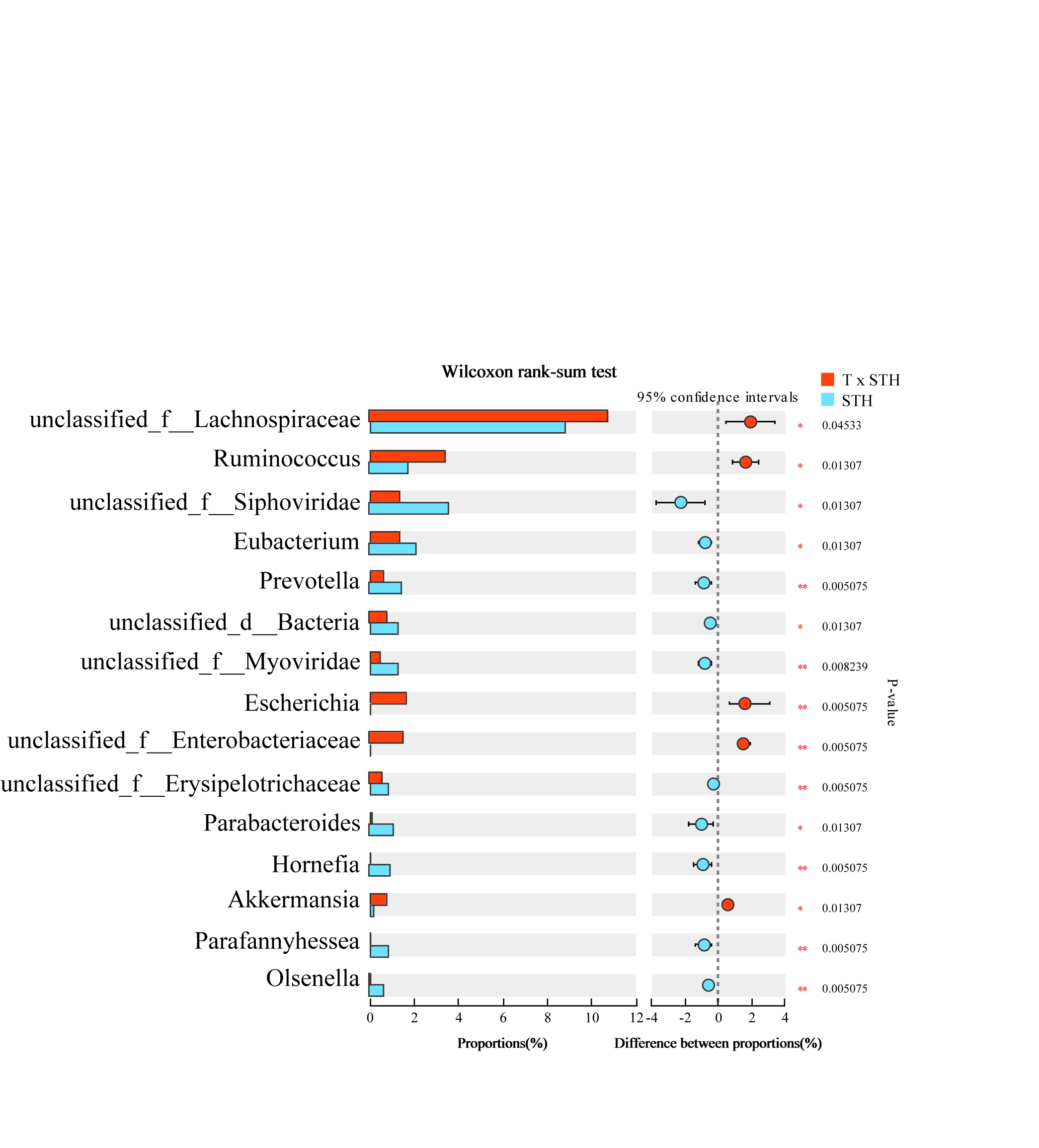

Supplement: SUPPLEMENTARY FIGURE S2 — Microorganisms that differed between groups at the genus level. [file Image_2.TIF]

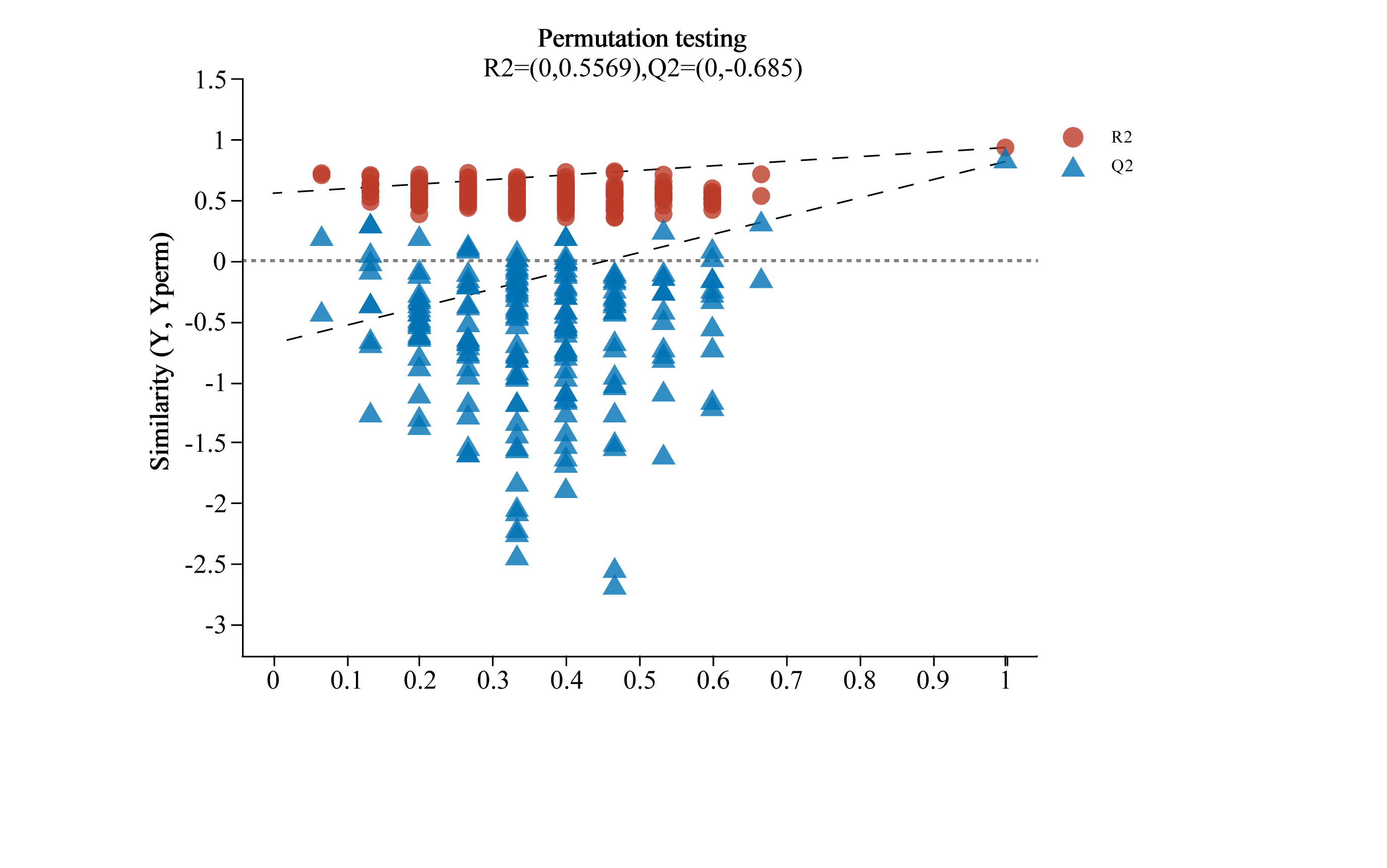

Supplement: SUPPLEMENTARY FIGURE S3 — Results of metabolite replacement test. [file Image_3.TIF]

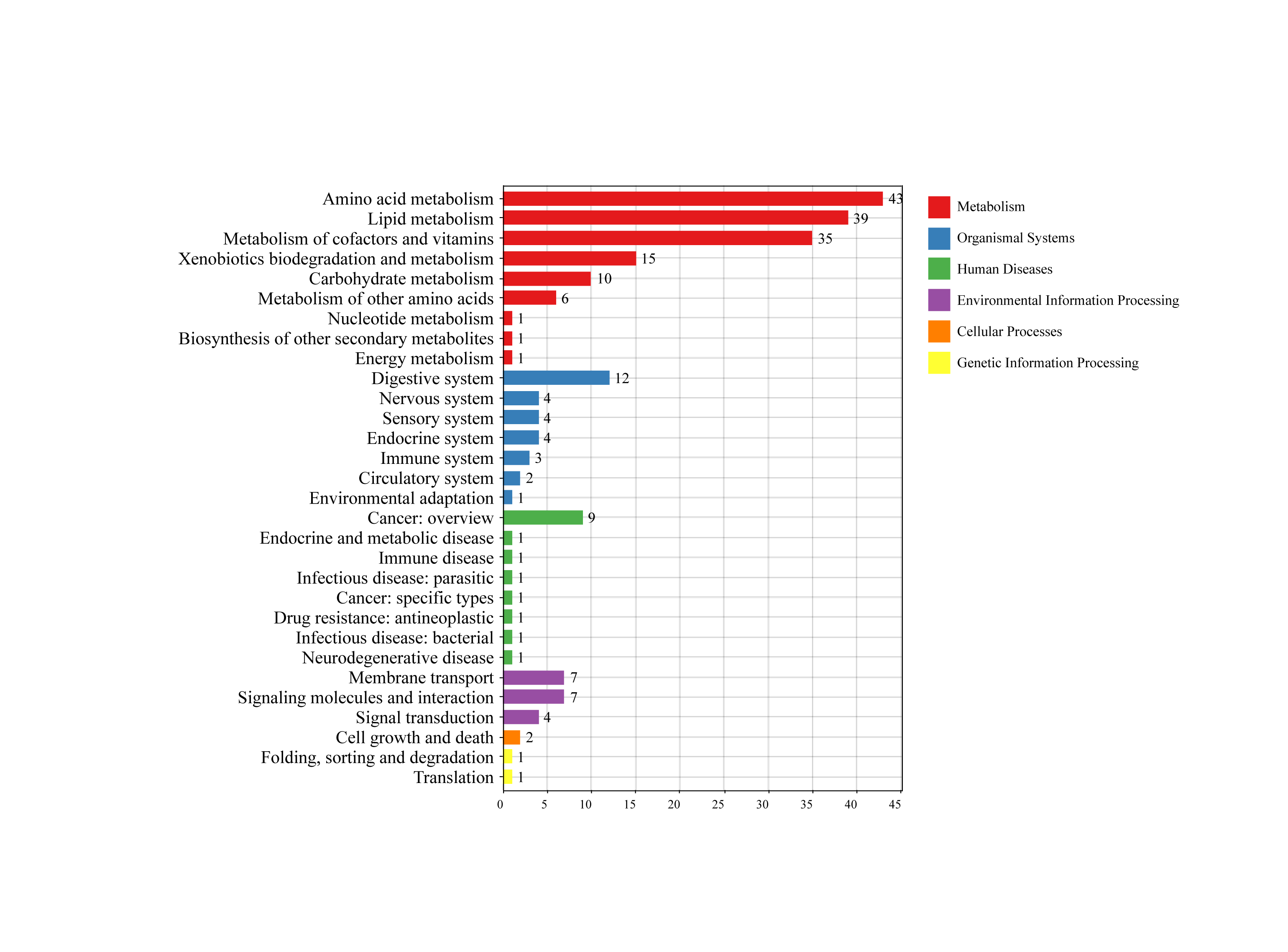

Supplement: SUPPLEMENTARY FIGURE S4 — KEGG pathway level 2 signaling pathway enriched by metabolites. [file Image_4.TIF]
